# Senescence evasion by MCF-7 human breast tumor-initiating cells

**DOI:** 10.1186/bcr2583

**Published:** 2010-06-02

**Authors:** Feridoun Karimi-Busheri, Aghdass Rasouli-Nia, John R Mackey, Michael Weinfeld

**Affiliations:** 1 Department of Oncology, University of Alberta and Department of Experimental Oncology, Cross Cancer Institute, 11560 University Avenue, Edmonton, Alberta T6G 1Z2, Canada; 2 NovaRx Corporation, Stem Cell Program, 6828 Nancy Ridge Drive, San Diego, CA 92121, USA

## Abstract

**Introduction:**

A subpopulation of cancer cells, tumor-initiating cells, is believed to be the driving force behind tumorigenesis and resistance to radiation and chemotherapy. The persistence of tumor-initiating cells may depend on altered regulation of DNA damage and checkpoint proteins, as well as a reduced propensity to undergo apoptosis or senescence.

**Methods:**

To test this hypothesis, we isolated CD24^-/low^/CD44^+ ^tumor-initiating cells (as mammospheres) from MCF-7 breast cancer cells grown in adherent monolayer culture, and carried out a comprehensive comparison of cell death and DNA damage response pathways prior to and after exposure to ionizing radiation in mammospheres and monolayer MCF-7 cells. Single and double-strand break repair was measured by single-cell gel electrophoresis. The latter was also examined by phosphorylation of histone H2AX and formation of 53BP1 and Rad51 foci. Apoptosis was quantified by flow-cytometric analysis of annexin V-binding and senescence was analyzed on the basis of cellular β-galactosidase activity. We employed the telomeric repeat amplification protocol to quantify telomerase activity. Expression of key DNA repair and cell cycle regulatory proteins was detected and quantified by western blot analysis.

**Results:**

Our data demonstrate that in comparison to the bulk population of MCF-7 cells (predominantly CD24^+^/CD44^+^), the MCF-7 mammosphere cells benefit from a multifaceted approach to cellular protection relative to that seen in monolayer cells, including a reduced level of reactive oxygen species, a more active DNA single-strand break repair (SSBR) pathway, possibly due to a higher level of expression of the key SSBR protein, human AP endonuclease 1 (Ape1), and a significantly reduced propensity to undergo senescence as a result of increased telomerase activity and a low level of p21 protein expression. No significant difference was seen in the rates of double-strand break repair (DSBR) between the two cell types, but DSBR in mammospheres appears to by-pass the need for H2AX phosphorylation.

**Conclusions:**

Enhanced survival of MCF-7 tumor-initiating cells in response to ionizing radiation is primarily dependent on an inherent down-regulation of the senescence pathway. Since MCF-7 cells are representative of cancer cells that do not readily undergo apoptosis, consideration of senescence pathways may play a role in targeting stem cells from such tumors.

## Introduction

Although considerable information has been amassed concerning potential risk factors and the genetic background of breast cancer, the etiology of the disease is still poorly understood [1]. Recent evidence led to the proposal that normal stem cells may be the key cells in a tissue or organ that undergo mutation and transformation giving rise to 'cancer stem cells' [2-5]. As normal stem cells are long-lived cells and the precursors to differentiated cells, DNA repair and mutation avoidance in these cells should be critical. Mutation and transformation of normal stem cells are most likely the result of DNA damage arising from exogenous and endogenous agents, including oxidative free radicals and dietary and environmental factors [6,7]. To counter such damage, cells possess a variety of multi-protein DNA repair pathways, each responsible for handling a class of DNA lesions [8].

Until recently, evidence to support a direct role for an altered DNA repair response in normal and cancer stem cells was limited and mostly confined to hematopoietic cells [9-11]. A study of bone marrow-derived mesenchymal stem cells, for example, identified a more efficient reactive oxygen species (ROS) scavenging capacity in these cells. Moreover, these stem cells exhibit active homologous recombination (HR) and nonhomologous end-joining (NHEJ) in the repair of double-stranded breaks to facilitate their radio-resistance [11]. Data from studies with murine embryonic stem cells indicate that these cells efficiently repair DNA damage [12-14], and that repair in embryonic stem cells may even be superior to that in differentiated embryoid bodies or embryonic fibroblasts [13]. The spontaneous mutation frequency in murine embryonic stem cells is also significantly lower than that in differentiated embryonic fibroblasts [15].

For cancer stem cells, mutation avoidance may be less crucial, but cell survival should be a dominant characteristic and may result in enhanced resistance to radiation and chemotherapeutic agents [4,16]. Thus, there is a clear need to identify the mechanisms that are involved in the maintenance of genome stability and cell survival in cancer stem cells. It has proven extremely difficult to culture sufficient numbers of stem cells from fresh solid tumor material for such studies. However, many established tumor cell lines possess a small fraction of self-renewing tumor-initiating (stem) cells that can form tumors from very few cells [5,17-19]. Studies with such glioma [20] and breast tumor-initiating cells [16,21,22], cultured as neurospheres and mammospheres, respectively, have provided some insights. In both cases the stem cells were observed to have elevated resistance to ionizing radiation, and additional data indicated enhanced DNA damage checkpoint activation [20] and repair capacity, as well as a marked reduction in the measured level of ROS following exposure to ionizing radiation [21], in comparison to the monolayer cell populations from which the stem cells were isolated.

To further explore the role of factors that safeguard genomic integrity and cell survival, we carried out a detailed comparison of single-strand break repair (SSBR) and double-strand break repair (DSBR), telomerase activity, and cell death in MCF-7 breast cancer cells and tumor-initiating cells isolated from the MCF-7 population as mammospheres [21,23]. To date most attention has focused on the apoptotic response of cancer stem cells exposed to ionizing radiation and other cytotoxic agents. Here we show for the first time that solid tumor cancer cell 'stemness' can also influence the senescence pathway.

## Materials and methods

### Cell culture and mammosphere preparation

The MCF-7 breast cancer cell line was obtained from the American Type Culture Collection (Manassas, VA, USA). The cells were cultured in 1:1 DMEM/nutrient mixture F12 supplemented with 10% fetal bovine serum, 50 units/ml penicillin, 50 µg/ml streptomycin, 2 mM L-glutamine, 0.1 mM nonessential amino acids and 1 mM sodium pyruvate, all from Gibco/BRL-Invitrogen (Carlsbad, CA, USA), and maintained at 37°C under 5% carbon dioxide in a humidified incubator. When cells approached confluency, the supernatant was collected and plated on ultralow attachment plates (Corning, Acton, MA, USA) in serum-free medium supplemented with basic fibroblast growth factor (Millipore, Billerica, MA, USA), epithelial growth factor and insulin (Sigma, St. Louis, MO, USA) as described previously [23]. Mammospheres, appearing as spheres of floating viable cells, were collected by centrifugation and dissociated with 0.05% trypsin followed by mechanical dissociation with a Pasteur pipette. Cells were then sieved sequentially through a 100 µm and a 40 µm cell strainer (Falcon, San Jose, CA, USA) and plated at low density.

### Flow cytometry and cell cycle analysis

Purified mammospheres were characterized by analysis of appropriate cell surface markers, CD24 and CD44, on a Coulter Epics flow cytometer (Becton Dickinson, San Jose, CA, USA). Briefly, 0.5 × 10^6 ^cells were mixed with fluorescently-tagged antibodies to the surface antigens (R-phycoerythrin-labelled anti-CD24 and fluorescein isothiocyante-labelled anti-CD44 antibodies; BD Pharmingen, San Diego, CA, USA) and incubated in the dark for 15 minutes at room temperature. Following incubation, cells were washed with PBS containing 1% FCS, centrifuged for five minutes at 2,500 rpm. After aspiration, approximately 50 μl of the cells were resuspended in PBS containing 1% FCS and underwent fluorescence activated cell sorter analysis immediately.

For cell cycle analysis, approximately 1 × 10^6 ^cells in an aliquot of 1 ml were fixed by the addition of 3 ml cold absolute ethanol. After one hour, cells were pelleted, washed twice in PBS and treated with propidium iodide/RNAse solution for 15 minutes at 37°C. Samples were placed in 12 × 75 mm Falcon tubes and analysed by a Coulter Epics flow cytometer.

### Clonogenic cell survival assay

Stock cultures of MCF-7 monolayer and mammosphere cells were trypsinized, resuspended in DMEM/F12 medium supplemented with 10% FCS, and plated in 60 mm tissue culture dishes at densities of 200 to 600 cells per dish. The cells, as a single-cell suspension, were then subjected to γ-radiation. After radiation, the cells were allowed to adhere to the plates and incubated at 37°C for 18 days, after which they were fixed and stained with crystal violet. Colonies consisting of 30 or more cells were counted and the surviving fraction (in comparison to unirradiated controls) was plotted as a function of dose using PRISM software (version 5.0 GraphPad, San Diego, CA, USA)

### Cell proliferation

Cellular survival of monolayer and mammospheres were measured by cell proliferation assay using CellTiter 96 AQueous One Solution Cell Proliferation Assay (Promega, Madison, WI, USA). For this assay, both monolayer and mammospheres were trypsinized and plated in a 96-well plate at a density of 2 × 10^3 ^cells per well. Monolayer cells were cultured in DMEM/F12 supplemented with 10% FCS and mammosphere media consisted of DMEM/F12 supplemented with 0.4% fetal serum albumin, 20 ng/ml basic fibroblast growth factor, 10 ng/ml epidermal growth factor, 5 μg/ml bovine insulin, and 4 μg/ml heparin. The cells were then subjected to increasing doses of γ- radiation (2 to 8 Gy) and incubated for five days at 37°C. Following incubation, 20 μl of CellTiter reagent (Promega, Madison, WI, USA) was added per well and after four hours of incubation at 37°C the absorbance was recorded at 490 nm and plotted verses radiation doses. All measurements were carried out in triplicate.

### Detection of reactive oxygen species

For the detection of highly ROS we employed an aminophenyl fluorescein dye-based kit (Cell Technology Inc, Mountain View, CA, USA) according to the manufacturer's protocol. Briefly, cells were rinsed in modified Hank's balanced salt solution (10 mM 4-(2-hydroxyethyl)piperazine -N'-(2-ethanesulfonic acid), 1 mM MgCl_2_, 2 mM CaCl_2_, 2.7 mM glucose) and aminophenyl fluorescein was added (to a final concentration of 10 μM) to approximately 1.5 × 10^6 ^cells. Cells were incubated for 30 minutes in the dark and then exposed to 1, 4, or 10 Gy γ-radiation (^60^Co Gammacell; Atomic Energy of Canada Limited, Ottawa, Canada) and plated at 3 × 10^5 ^cells per 96-well plate. The fluorescence was immediately measured by excitation at 485 nm and reading emission at 515 nm using a fluorescence plate reader (Cell Technology Inc., Columbia, MD, USA).

### Single-cell gel electrophoresis (Comet assay)

At various times after irradiation of the cells, approximately 1 × 10^5 ^cells were trypsinized and mixed with 1.0 % molten low melting point agarose at 42°C at a 1:10 ratio (10 μl cells per 100 μl of agarose). The mixture was then spread on a glass slide (Trevigen, Gaithersburg, MD, USA). Following solidification of the agarose, slides were immersed in prechilled lysis solution (2.5 M NaCl, 100 mM Na_2_EDTA, pH 10, 10 mM Tris base, 1% SDS, 1% Triton X-100), followed by incubation on ice for 40 minutes and immersion in an alkaline solution (300 mM NaOH, 1 mM Na_2_EDTA) for 30 minutes at room temperature. Slides were then placed in an electrophoresis apparatus, filled with fresh alkaline solution, and run at 1 V/cm and approximately 300 mA at 4°C for 40 minutes. The slides were washed in 70% ethanol for five minutes and the DNA was stained with SYBR Green I (Molecular Probes-Invitrogen, Carlsbad, CA, USA) and viewed with an AxioScope 2 fluorescence microscope (Carl Zeiss, Toronto, ON, Canada). For each data point we visualized and analyzed a minimum of 100 random cell images based on the 'comet' category shown by Collins [24]. (We have changed the numbering 0 to 4 to 1 to 5).

For the neutral non-denaturing comet assay the cells were lysed as described above followed by rinsing in 50 ml 1 × TBE (89 mM Tris base, 89 mM boric acid, 2 mM EDTA) buffer. The slides were then subjected to electrophoresis at 1 volt/cm for 40 minutes in a horizontal electrophoresis apparatus, followed by fixation in 70% ethanol for five minutes and air dried. The slides were then stained and scored as for alkaline comet.

### Preparation of cell-free extract, immunoblotting, and immunostaining

For preparation of cell lysate we followed a previously published method [25]. For western blotting and immunohistochemistry standard protocols currently applied to breast stem cells were used [26,27]. Antibodies to the following proteins were obtained commercially: PCNA (monoclonal, sc-25280), XRCC1 (polyclonal, sc-11429), Chk1 (polyclonal, sc-7898), Chk2 (polyclonal, sc-9064), p21 (monoclonal, sc-6246), pRb (monoclonal, sc-103), phospho-Chk1 (polyclonal, sc-17922R), Cdc25A (polyclonal, sc-7157), MDM2 (monoclonal, sc-5304), p53 (monoclonal, sc-126), E2F1 (monoclonal, sc-251), and actin (polyclonal, sc-1616) from Santa Cruz Biotech (Santa Cruz, CA, USA); ataxia telangiectasia mutated (ATM; polyclonal, NB 100-104), apurinic/apyrimidinic endonuclease 1 (APE1; monoclonal, NB 100-116) from Novus Biologicals (Littleton, CO, USA); phospho-Chk2 (polyclonal, 2661), and phospho-p53 (sampler kit, #9919) antibodies from Cell Signaling (Boston, MA, USA); phospho-ATM (polyclonal, ab2888) and phospho-DNA-dependent protein kinase catalytic subunit (DNA-PKcs; polyclonal, ab18192) from Abcam (Cambridge, MA, USA); PNK (monoclonal, MAB005) from CytoStore (Calgary, AB, Canada); epithelial specific antigen (FITC-labeled monoclonal, MM-1014) from Immunobiosciences (Raleigh, NC, USA). Polyclonal anti-Ku80 antibody was a gift from Dr. Susan Lees-Miller (University of Calgary, Calgary, AB, Canada), antibody to polymerase-β was a gift from Dr. Sam Wilson (National Institute of Environmental Health Sciences, Research Triangle Park, NC, USA), and monoclonal antibody to ING1 was kindly provided by Dr. Karl Riabowol (University of Calgary, Calgary, AB, Canada). Proteins on western blots were visualized by enhanced chemiluminescence (Amersham Pharmacia Biotech, Arlington Heights, IL, USA) and X-Omat K film (Eastman Kodak Co., Rochester, NY, USA). Films were scanned as image files and the optical densities of the bands were quantified using ImageJ software version 1.33u (NIH, Bethesda, MA, USA).

Statistical analysis by two-tailed Student's t-test was performed with Microsoft Excel software (Microsoft Corp, Redmond, WA, USA).

For mammosphere immunostaining, we used a Cytospin centrifuge (Thermo Shandon, Pittsburgh, PA, USA) to prepare thin-layer mammospheres slides. Cells were dried and fixed by paraformaldehyde. After blocking with 5% milk powder, the Cytospin smears were stained with appropriate primary and secondary antibodies.

### Imaging of γH2AX, 53BP1 and Rad51 foci

We monitored the level of H2AX phosphorylation before and after ionizing radiation by the modified protocol of Furuta and colleagues [28]. Briefly, 1 × 10^5 ^MCF-7 cells were seeded onto each glass coverslip inside 35 mm tissue culture dishes. For mammospheres, however, we used a Cytospin to prepare thin-layer mammosphere slides. After incubation overnight at 37°C in a carbon dioxide incubator, the cells were exposed to 1 or 10 Gy γ-radiation and then incubated for different times at 37°C. The cells were then fixed in 2% paraformaldehyde in PBS for five minutes, followed by washes with PBS and 50:50 methanol/PBS solution, and permeabilization with 100% methanol for 20 minutes at -20°C. Cells were then blocked with 5% milk powder at room temperature and then exposed to a monoclonal antibody to anti-phospho-histone H2AX (Ser-139) (Upstate, Charlottesville, VA, USA) for one hour in the dark. After washing the coverslips with PBS, Alexa Fluor 488-labelled goat anti-mouse secondary antibody (Molecular Probes-Invitrogen, Carlsbad, CA, USA) was added to the coverslips and incubated at room temperature for one hour in the dark. The coverslips were again washed twice with PBS and PBS/Tween 20 and finally rinsed with water. The coverslips were mounted on a microscope slide using 95% glycerol in PBS containing 3 µg/ml 4',6-diamidino-2-phenylindole. Slides were stored at 4°C in the dark. Phosphorylated H2AX foci were viewed with a LSM510 laser-scanning confocal microscope (Carl Zeiss, Toronto, ON, Canada) mounted on a Axiovert 100 M microscope (Carl Zeiss, Toronto, ON, Canada). Images were taken with a ×40 (NA 1.3) objective, following the Nyquist sampling requirement, with the same instrument settings for different slides. The average integrated intensity per nucleus was determined by using METAMORPH OFFLINE 6.1 software (Universal Imaging, Downingtown, PA, USA), as described previously [29].

The same procedure was followed to monitor 53BP1 and Rad51 foci. For the former we used an anti-53BP1 rabbit polyclonal antibody (ab36823) from Abcam (Cambridge, MA, USA), and for the latter an anti-Rad51mouse monoclonal antibody (ab213) from Abcam (Cambridge, MA, USA).

### Real-time quantitative RT-PCR

Total RNA was extracted from 2 × 10^6 ^cells using TRIZOL Reagent (Invitrogen, Carlsbad, CA, USA) as per the manufacturer's instructions. RNA concentration was determined spectrophotometrically with a Life Science UV/Vis Spectrophotometer (Beckman Coulter Du 730, Brea, CA, USA).

The quantitative RT-PCR assay was performed on a 7900HT Real-Time PCR system (AB Applied Biosystem, Carlsbad, CA, USA). Total RNA (0.5 μg) from each sample and standard was amplified using Platinum SYBR Green quantitative PCR SuperMix-UDG (Invitrogen, Carlsbad, CA, USA) according to the manufacturer's protocol. The primer sequences for APE1 gene were 5'-GCTGCCTGGACTCTCTCATC-3' (sense), 5'-GCTGTTACCAGCACAAACGA-3' (antisense) to generate a 180 bp product. The primer sequences for ING1b were 5'-CAACAACGAGAACCGTGAGA-3' (sense) and 5'-GAGACCTGGTTGCACAGACA-3' (antisense) to give a 195 bp product. For GAPDH, as control, the primer sequences were 5'-GTCTCCTCTGACTTCAACAGCG-3' (sense) and 5'-ACCACCCTGTTGCTGTAGCCAA-3' (antisense) to yield a 130 bp product. PCR conditions were as follows: 50°C for 6 minutes, 95°C for 10 minutes and 40 cycles of 95°C for 15 seconds and 60°C for 30 seconds followed by 2 minutes incubation at 60°C. The cycle threshold value for each gene was normalized by subtracting the cycle threshold value of the GAPDH control gene, and then the relative fold increase in transcript level was calculated.

### Telomerase assay

The telomerase assay was performed with a TRAPeze Telomerase Detection kit (Chemicon, Temecula, CA, USA) using non-radioactive detection. Whole cell extracts were prepared from 10^6 ^unirradiated and irradiated MCF-7 monolayer cells and 10^6 ^unirradiated and irradiated mammosphere cells in CHAPS lysis buffer provided with the kit according to the manufacturer's instruction. Samples of extract equivalent to 10^4 ^cells were used in each assay. Following incubation with cell extract and PCR amplification, the samples were analyzed by polyacrylamide gel electrophoresis and ethidium bromide staining. The optical densities of the scanned bands were quantified using ImageJ software version 1.33u (NIH, Bethesda, MD, USA). Statistical analysis by one-tailed Student's t-test was performed with Microsoft Excel software (Microsoft Corp, Redmond, WA, USA).

### Apoptosis and senescence-associated β-Gal assays

For the detection of apoptosis, unirradiated cells, or cells exposed to 4 Gy γ-radiation (^60^Co Gammacell; Atomic Energy of Canada Limited, Ottawa, Canada) three days prior to collection, were harvested and analyzed by flow cytometry for the induction of apoptosis using the BD Pharmingen-Annexin V: FITC Apoptosis Detection Kit (San Jose, CA, USA) according to the manufacturer's instructions.

Induction of senescence was observed by morphological changes detectable under light microscopy, and by looking at cellular β-galactosidase activity [30], a known marker of senescent cells, using a Senescence Galactosidase Staining Kit (Cell Signaling, Beverly, MA, USA) according to the manufacturer's protocol. As mammospheres are non-adherent cells we used a Shandon Cytospin centrifuge (Waltham, MA, USA) to spread cells over the glass slides prior to fixation and staining.

## Results

### MCF-7 mammosphere isolation, radiation response and ROS content

Actively growing CD24^-/low^/CD44^+ ^non-adherent mammospheres were isolated from monolayer cultures of MCF-7 breast cancer cells according to published procedures [23], with some minor modifications [see Additional file 1]. We typically observed about 50% of mammosphere cell population to have a CD24^-/low^/CD44^+ ^expression compared with less than 2% in the monolayer cell population. We also examined expression of epithelial specific antigen and, in agreement with Fillmore and Kuperwasser [18], found the majority of both the adherent population and mammospheres expressed high levels of epithelial specific antigen (data not shown). The mammosphere cell population was shown to be predominantly (about 80%) in G0/G1 with approximately 10% of cells in S and 10% in G2/M [see Additional file 2]. By comparison, the monolayer population was approximately 60% G0/G1, 25% S and 15% G2/M.

**Figure 1:**
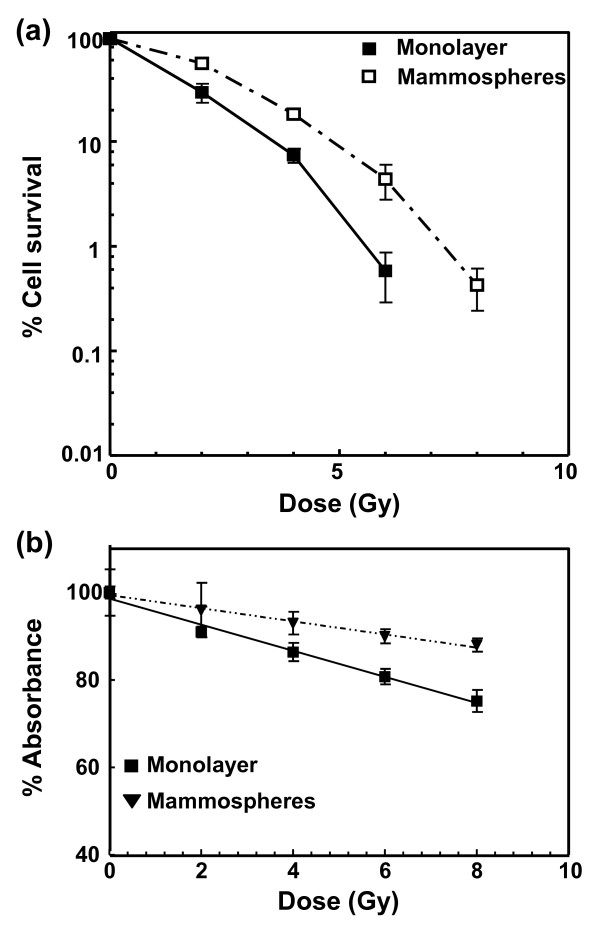
**Radiation response of MCF-7 monolayer and mammospheres cells**. **(a) **Clonogenic survival assay - MCF-7 monolayer and mammospheres were trypsinized, irradiated as single-cell suspensions with increasing doses of ^60^Co γ-radiation, and then plated. After18 days of incubation, the colonies (consisting of more than 30 cells) were fixed, stained with crystal violet and counted. **(b) **Cell proliferation assay - cells were plated in a 96-well plate, irradiated and incubated for five days. Cells were exposed to 3-(4,5-dimethylthiazol-2-yl)-5-(3-carboxymethoxyphenyl)-2-(4-sulfophenyl)-2H-tetrazolium (MTS, inner salt) for four hours and absorbance was measured at 490 nm. Absorbance was normalized to unirradiated controls. Error bars represent the mean ± standard error of the mean from three independent experiments.

Phillips and colleagues [21] have previously shown that the MCF-7 mammosphere population was less sensitive to ionizing radiation than the monolayer cells. We observed a similar radiation resistance in our preparation of mammospheres based on measurement of cell survival and cell proliferation (Figure 1). The colony-forming assay used to monitor cell survival entailed irradiation of both types of cells as single-cell suspensions, after which the cells were allowed to adhere and cultured under identical conditions. For cell proliferation the monolayer cells were grown under adherent conditions, while the mammospheres remained in suspension after irradiation.

**Figure 2:**
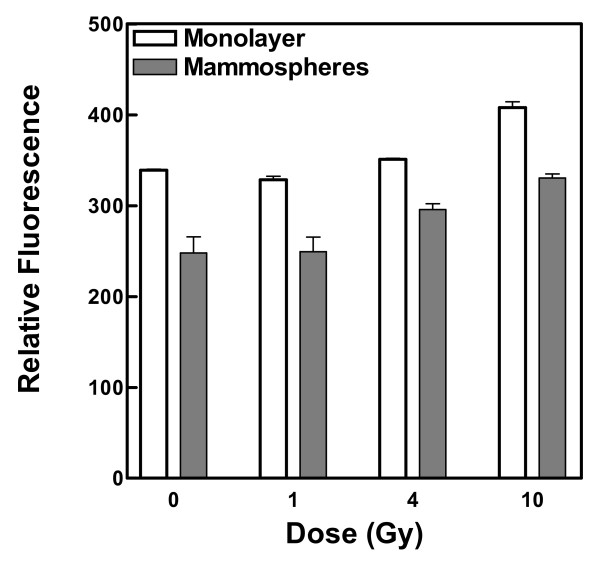
**Relative content of reactive oxygen species in unirradiated and irradiated MCF-7 monolayer and mammosphere cells**. Cells were incubated with aminophenyl fluorescein, exposed to 0, 1, 4 or 10 Gy ^60^Co γ-radiation, and immediately afterwards the relative fluorescence of each sample was measured by excitation at 485 nm and emission at 515 nm. Error bars represent the mean ± standard error of the mean from three independent experiments.

Cell death and mutation can result from exposure to endogenous and exogenous oxidative agents that generate base damage and single-strand breaks and, less frequently, double-strand breaks. We therefore measured ROS in the mammospheres and monolayer cells both before and after irradiation (Figure 2) and found a significantly lower level of ROS in mammospheres, in agreement with others [21]. In our study the reduced level of ROS appears to be primarily attributable to lower production of ROS in mammosphere cells rather than increased neutralization, because cell irradiation increased ROS by the same amount in both cell types.

### Repair of radiation-induced strand breaks

**Figure 3:**
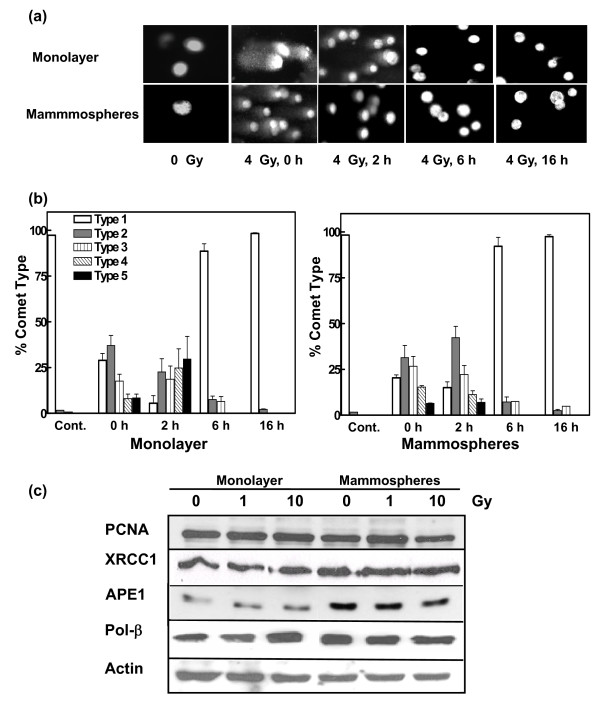
**Analysis of single strand break repair in MCF-7 monolayer and mammosphere cell populations**. **(a) **Cells were exposed to 4 Gy ^60^Co γ-radiation and the relative degree of single-strand breakage (SSB) was determined by alkaline single-cell gel electrophoresis (comet assay) immediately after exposure and at the times indicated after exposure. **(b) **The 'comets' (n of about 100) were categorized according to the NIH LISTSERV (Comet Assay Interest Group web site) in which type 1 comets display the least DNA damage and type 5 the most. The error bars represent the mean ± standard error of the mean in both panels. The comets of the unirradiated cells are labeled Cont. **(c) **Expression of proteins involved in SSB repair in response to ionizing radiation. Lysates were prepared from unirradiated cells and from cells harvested one hour after exposure to 1 or 10-Gy ^60^Co γ-radiation and analyzed by immunoblotting with antibodies against several SSB repair proteins. α-Actin served as a loading control.

The capacity to respond to ROS-induced DNA damage is also a critical determinant in cell survival. A comparison of SSBR, determined by single-cell gel electrophoresis (comet assay) under alkaline conditions, indicated more rapid repair at early times (two hours) in the CD24^-/low^/CD44^+ ^MCF-7 cells than the monolayer cells following irradiation with 4 Gy (Figures 3a and 3b). The data are similar to those obtained with glioma stem cells [20]. An analysis of the expression of a panel of key SSBR proteins (Figure 3c) indicated similar levels of expression between mammospheres and monolayer cells with the notable exception of APE1, which was expressed at an elevated level (about two-fold) in unirradiated as well as irradiated mammospheres (Table 1). APE1 cleaves DNA at abasic sites and processes 3'-strand break termini, particularly the removal of 3'-phosphoglycolate groups [31]. It is required for mammalian cell survival and down-regulation leads to the accumulation of abasic sites and apoptosis [32,33]. Real-time quantitative RT-PCR indicated an approximately 10-fold increase (based on cycle threshold) in APE mRNA in mammospheres than the monolayer cells (data not shown), indicating that the increased expression of APE1 seen in mammospheres is at least partly regulated at the transcriptional level.

**Table 1:** Relative expression of DNA repair and cell cycle proteins in MCF-7 monolayer and mammosphere populations

Protein	Dose(Gy)	Normalized band intensity (n = 3)		*P* value
			
		Monolayer	Mammosphere	
APE1	0	1.00	2.10	0.024
	1	1.02	1.92	0.052
	10	1.07	1.84	0.017

ATM	0	1.00	1.99	0.018
	1	1.13	2.23	0.011
	10	1.62	1.99	0.088

p21	0	1.00	0.04	0.001
	1	1.16	1.04	0.207
	10	1.17	1.11	0.310

Chk1	0	1.00	2.17	0.003
	1	1.03	2.06	0.018
	10	1.05	2.31	0.052

phospho-Chk1	0	1.00	0.92	0.116
	1	1.13	0.80	0.027
	10	1.08	0.85	0.006

Chk2	0	1.00	2.39	0.001
	1	0.92	2.46	0.001
	10	0.93	2.51	0.005

phospho-Chk2	0	1.00	0.69	0.034
	1	0.91	0.64	0.030
	10	0.84	0.91	0.180

APE1, apurinic/apyrimidinic endonuclease 1; ATM, ataxia telangiectasia mutated; phospho-Chk1, Chk1 phosphorylated at Ser345; phospho-Chk2, Chk2 phosphorylated at Thr68.

**Figure 4:**
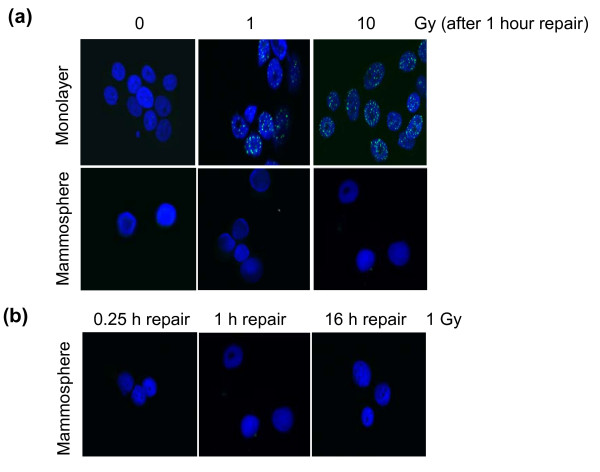
**Radiation induced H2AX phosphorylation in MCF-7 monolayer and mammosphere cells**. **(a) **Unirradiated cells or cells exposed to 1 or 10 Gy γ-radiation and then incubated for one hour at 37°C, or **(b) **cells exposed to 1 Gy and incubated at 37°C for different times, were fixed, permeabilized and immunostained with an antibody to γH2AX and counter-stained with 4',6-diamidino-2-phenylindole (DAPI). H2AX phosphorylation was clearly visible as characteristic fluorescent foci in the irradiated monolayer cells, but no foci were detectable in the irradiated mammosphere cells.

**Figure 5:**
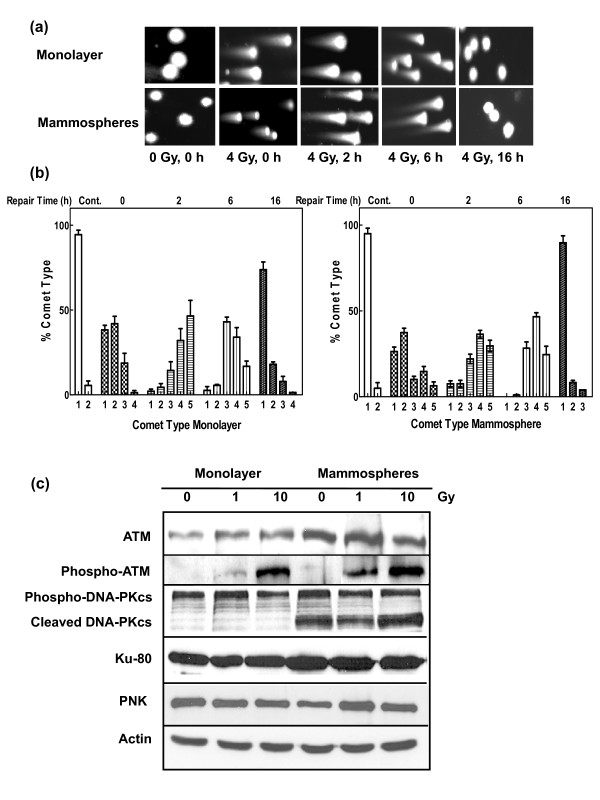
**Analysis of double strand break repair in MCF-7 monolayer and mammosphere populations**. **(a) **Cells were exposed to 4 Gy ^60^Co γ-radiation and the relative degree of double-strand breakage (DSB) was determined by the comet assay under neutral conditions immediately after exposure and at the times indicated after exposure. **(b) **The 'comets' (n of about 100) were categorized according to the NIH LISTSERV (Comet Assay Interest Group web site) in which type 1 comets display the least DNA damage and type 5 the most. The error bars represent the mean ± standard error of the mean in both panels. The comets of the unirradiated cells are labeled Cont. **(c) **Expression of proteins involved in the NHEJ pathway of DSB repair in response to increasing doses of ionizing radiation. Lysates were prepared from unirradiated cells and from cells harvested one hour after exposure to 1 or 10-Gy ^60^Co γ-radiation and analyzed by immunoblotting with antibodies against several DSB repair proteins. Phospho-ATM and phospho-DNA-PKcs refer to phosphorylation of these proteins at Ser1981 and Ser2056, respectively. Actin served as a loading control.

We next analyzed the rate of DSBR in the two cell types. Initially, we attempted to monitor DSBR by following the formation and disappearance of γ-H2AX foci. However, unlike the MCF-7 monolayer cells, the mammospheres failed to generate observable foci (Figure 4), an observation that concurs with Phillips and colleagues [21]. (These authors had used a flow cytometric approach to monitor γ-H2AX, so we were keen to confirm their surprising observation by more conventional cell imaging. We also expanded the time of observation from the one-hour post-irradiation time point chosen by Phillips and colleagues to 0.25 to 16 hours post-irradiation). We therefore followed double-strand break formation and repair by single-cell gel electrophoresis under neutral conditions, which indicated that as expected similar levels of double-strand breaks are generated in the two cell types and they are repaired at similar rates (Figures 5a and 5b). Thus DSBR in mammospheres appears to bypass the formation of γ-H2AX foci. Phosphorylation of H2AX plays an important role in both NHEJ and HR repair pathways, partly as a result of recruitment of other repair proteins to the damaged sites, although it is dispensable for initial damage recognition in NHEJ [34,35]. (As about 90% of the mammospheres were in G0/G1 and S-phase cells the predominant DSBR pathway would be predicted to be NHEJ). Furthermore, other DSBR pathways, such as single-strand annealing [36] and an XRCC1/DNA ligase III-dependent pathway [37], probably do not require H2AX phosphorylation. However, evidence that mammospheres, such as the monolayer cells, employ both NHEJ and HR was indicated by the formation of radiation-induced 53BP1 and Rad51 foci [see Additional file 3]. The former protein is principally associated with the NHEJ pathway [38], while the latter is involved in HR [39]. With both cell types, we observed a maximum in 53BP1 and Rad51 focus formation at two hours post-irradiation and a return to near background levels by 16 hours.

An examination of the expression of ATM and DNA-PKcs, the kinases primarily responsible for the phosphorylation of histone H2AX [34], revealed differences between the two cell types (Figure 5c). Firstly, unirradiated and irradiated mammospheres expressed higher levels of ATM (Table 1) and phospho-ATM (Ser1981) following irradiation. Secondly, examination of activated (phosphorylated) DNA-PKcs (phosphorylation at Ser2056) indicated a considerable proportion of the DNA-PKcs in the mammospheres appeared to have undergone cleavage. Similar proteolysis of phosphorylated DNA-PKcs has previously been reported in cells undergoing apoptosis [40] but, as discussed below, the level of apoptosis in the unirradiated and irradiated mammospheres was negligible. It is not known if the cleaved phosphorylated DNA-PKcs acts in a dominant negative fashion to inhibit phosphorylation of H2AX. The level of expression of other NHEJ proteins examined, Ku80 and PNK, were not grossly different in the two cell types (Figure 5c).

### MCF-7 mammospheres display reduced cellular senescence

**Figure 6:**
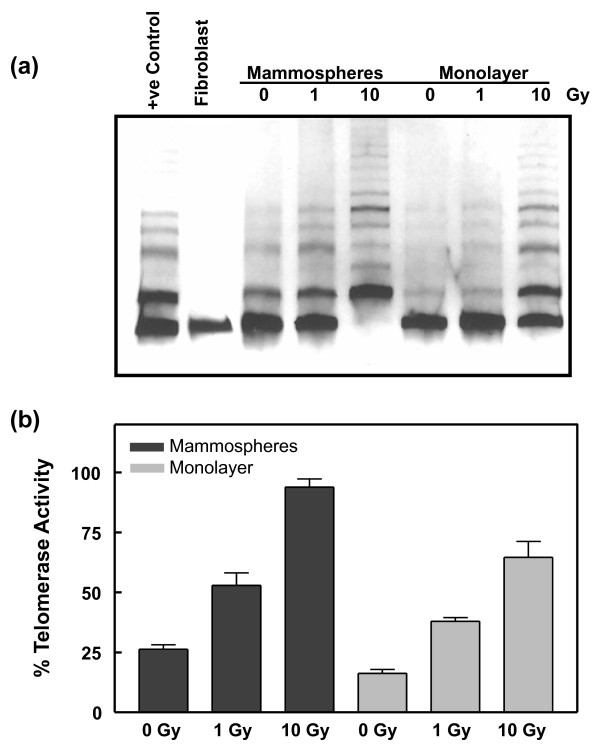
**Telomerase activity in MCF-7 monolayer and mammosphere cells**. **(a) **Telemorase activity was determined by Telomeric Repeat Amplification Protocol (TRAP) analysis. Cell extracts were prepared from unirradiated and irradiated MCF-7 monolayer and mammospheres cells. The assay involves the addition, mediated by telomerase in the cell extract, of a number of telomeric repeats onto the 3' end of an oligonucleotide substrate, which is then subjected to amplification by PCR. When run on a 10 to 12% native polyacrylamide gel, the telomerase-extended primer appears as a ladder of 6 bp increments. The lowest band on the gel shows the 36 bp internal PCR control. (The absence of this band in the lane showing results with 10-Gy irradiated mammospheres is due to excessively high telomerase activity because amplification of the TRAP products and the internal control is semi-competitive.) The positive (+ve) control lane shows the results using a telomerase positive cell extract provided by the manufacturer of the assay kit, while a cell extract prepared from human fibroblasts (CRL 2322) served as a negative control. **(b) **Quantification of telomerase activity. The mean values and standard deviations were calculated from three individual determinations at each dose. The difference in activity between the monolayer and mammosphere populations at each dose were statistically significant (*P *< 0.05, Student's t-test).

As the decreased level of ROS and more rapid SSBR appeared unlikely to sufficiently account for the relative radioresistance exhibited by mammospheres [21,22], we went on to examine cell death pathways in the MCF-7 populations, paying particular attention to cellular senescence because MCF-7 cells are not prone to apoptosis due to a lack of caspase-3 activation [41]. Replicative senescence is considered a process triggered by telomere shortening that limits the lifespan of normal cells, but it may also serve as a block to tumorigenesis in premalignant cells [42]. Ionizing radiation and similar cytotoxic agents can induce stress-induced premature or accelerated senescence in normal and tumor cell lines [43,44]. Telomerase, which is expressed in stem cells and in cancer cells [45], maintains telomere length, thereby preventing replicative senescence. The enzyme may also protect against premature or accelerated senescence by protecting chromosome ends from nucleolytic degradation [46,47]. Using the Telomeric Repeat Amplification Protocol to measure telomerase activity in monolayer and mammosphere cells (Figure 6a), we observed that both cell types displayed telomerase activity, which increased after irradiation with 1 and 10 Gy. Telomerase activity is revealed by the presence of the extended oligonucleotides migrating slower than the 36 bp primer. However, the telomerase activity in the mammospheres was statistically significantly more robust than in the monolayer population (Figure 6b).

**Figure 7:**
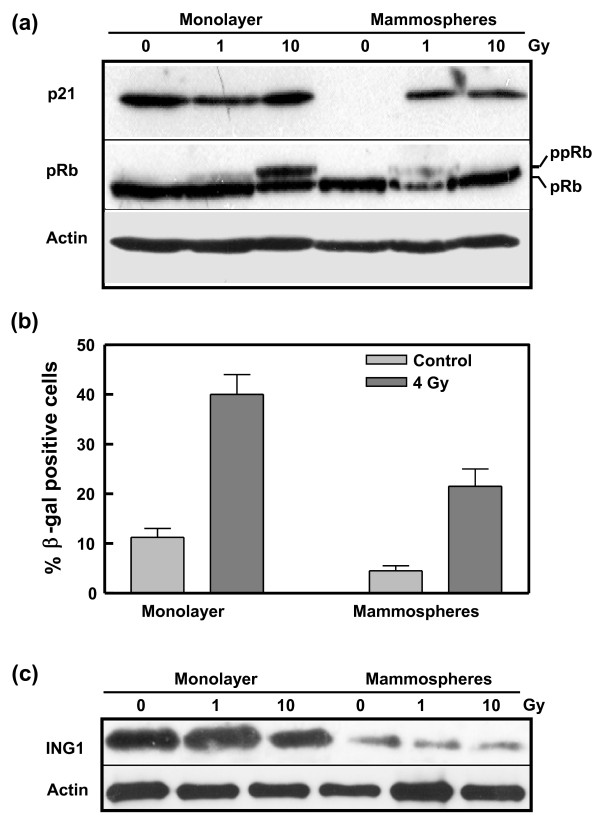
**Senescence-related factors in MCF-7 monolayer and mammosphere populations**. **(a) **Expression of key proteins involved in the cellular senescence pathway in response to increasing doses of ionizing radiation. Lysates were prepared from unirradiated cells and from cells harvested one hour after exposure to 1 or 10-Gy ^60^Co γ-radiation and analyzed by immunoblotting with antibodies against p21 and pRb. ppRb indicates phosphorylated pRb. Actin served as a loading control. **(b) **Senescence was measured in unirradiated (control) cells and irradiated (4 Gy, 3 days post-irradiation) MCF-7 monolayer and mammosphere cells by staining for β-galactosidase activity. Cells (n of more than 100) were counted independently by two individuals. The error bars represent the mean ± standard error of the mean. **(c) **Expression of ING1 protein. Lysates were prepared as described for Figure 6a. Western blots were performed with a mixture of four monoclonal antibodies against a domain common to ING1a, ING1b and ING1c. Based on the apparent molecular weight of the observed band (about 35 kDa), the protein was identified as ING1b.

An examination of other key proteins involved in the regulation of senescence revealed that the mammosphere cells, in comparison to the monolayer cell population, expressed markedly lower levels of the senescence signaling protein, p21, especially in unirradiated cells (Figure 7a). In the case of pRb there was a notable reduction in total protein following irradiation with 1 Gy and also a lower degree of phosphorylation after 10-Gy irradiation of the mammospheres. Inactivation of p21 and pRb is required to prevent senescence [48], and indeed analysis of senescence based on cellular β-galactosidase activity revealed that the percentage of senescent mammospheres is approximately half that of the monolayer population both prior to irradiation and three days after irradiation with 4 Gy (Figure 7b). As p21 is associated with telomere shortening and replicative senescence, it is perhaps not surprising that its expression is negligible in the unirradiated stem cell population. pRb, on the other hand, has been shown to regulate stress-induced senescent arrest in a p16-dependent pathway. However, in agreement with others [49] we found that MCF-7 cells fail to express p16.

Additional confirmation of the reduced tendency of the mammospheres to undergo senescence was provided by an analysis of the expression of ING1. Elevated expression of this tumor suppressor protein is strongly associated with increased replicative senescence [50]. Although there was no marked response to the radiation, it is clear from Figure 7c that the MCF-7 monolayer cells display a more than five-fold higher (*P *< 0.003) level of ING1 protein than the mammosphere cells. The enhanced level of ING1 protein was not reflected by an increased level of transcription as measured by quantitative RT-PCR (data not shown), suggesting that the protein in the monolayer cells may be stabilized or undergo slower turnover. The only ING1 isoform detected in these cells was ING1b. Interestingly, loss of expression of ING1b in breast tumors has been found to be associated with more poorly differentiated tumors and therefore may be indicative of poor prognosis [51].

We also observed a smaller percentage of apoptotic/necrotic cells (based on annexin V-FITC binding) in the unirradiated mammosphere population, despite the cleavage of DNA-PKcs, than in monolayer populations (2.8 vs 5.3%). Little difference was observed in these numbers after irradiation (data not shown) presumably because MCF-7 cells do not readily undergo apoptosis [41].

### Expression of DNA damage/cell cycle response proteins

**Figure 8:**
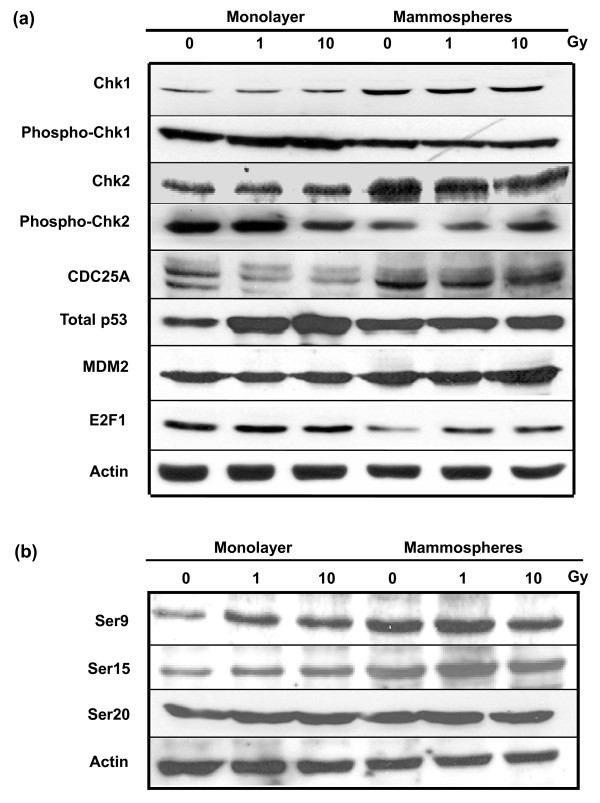
**Expression of DNA damage/cell cycle response proteins in unirradiated and irradiated MCF-7 cells**. **(a) **Lysates were prepared from unirradiated monolayer and mammosphere cells and from cells harvested one hour after exposure to 1 or 10-Gy ^60^Co γ-radiation and analyzed by immunoblotting. Phospho-Chk1 and phospho-Chk2 indicate phosphorylation of Chk1 and Chk2 at Ser345 and Thr68, respectively. Actin served as a loading control. **(b) **Phosphorylation of p53 at various amino acids. Phosphorylation at serines 6, 9, 37, 46, and 392 was negative (data not shown).

In light of our data indicating reduced expression levels of p21 and reduced phosphorylation of H2AX and pRb in mammospheres, we probed the status of other proteins involved in cell cycle and cell death responses to oxidative stress (Figure 8) downstream of ATM. The signal mediators, Chk1 and Chk2, reveal a similar pattern of expression and modification. In both cases, the level of total protein was higher in the unirradiated and irradiated mammospheres than monolayer cells, but the degree of phosphorylation (Chk1 Ser345 and Chk2 Thr68) was considerably lower. Phosphorylated Chk2, in turn, destabilizes Cdc25A by phosphorylation and targeting it for ubiquitination [52]. Consistent with a lower level of phosphorylated Chk2 in mammospheres, we observed a more pronounced signal for Cdc25A in these cells. On the other hand, Chk2 stabilizes E2F1 by phosphorylation of the latter protein [53]. We found mammospheres to contain moderately less E2F1 than the monolayer cells.

Expression of p53 in the MCF-7 monolayer population in response to irradiation followed the anticipated pattern, that is there was a dose-dependent increase in protein accumulation with elevated phosphorylation of serines 9 and 15. In the mammospheres, however, although the level of p53 in the unirradiated cells was higher than in the monolayer population, there was no clearly discernable increase in p53 in response to irradiation with 1 or 10 Gy. Furthermore, p53 in the unirradiated mammospheres was shown to be more highly phosphorylated at serines 9 and 15, and again this did not alter markedly with dose. Thus not only does the phosphorylation of p53 differ between the two cell types, but in addition the phosphorylation of p53 differs from the phosphorylation of H2AX, Chk1 and Chk2, despite their potential dependence on ATM. It suggests that either ATM preferentially phosphorylates p53 or that the levels of phosphorylation may reflect the relative activities of phosphatases such as Wip1 known to dephosphorylate Chk2 at Thr68 in a p53-dependent manner and act in a negative feedback loop between the two proteins [54]. Intriguingly, the pattern of p53 expression and phosphorylation in mammospheres and monolayer cells bears a resemblance to recent findings with p21-/- and wild type HCT116 colon cancer cells [55], that is high constitutive expression of p53 (and Chk2) and elevated phosphorylation of Ser15 in the untreated p21-/- cells and no change in either expression or phosphorylation in response to oxidative damage induced by chromium.

## Discussion

The data presented here demonstrate that mammospheres, as a representative of epithelial mammary cancer progenitors, are distinct from their differentiated MCF-7 cells. It has been previously shown by others that MCF-7 mammospheres are more resistant to ionizing radiation than the monolayer cells and have lower ROS content in unirradiated and irradiated cells [21,22], and we have corroborated their findings. Phillips and colleagues [21] also ascertained that the observed effects were not the result of differences in the growth factor content of the media in which the two cell types were cultured. In our laboratory, the clonogenic survival assays of the two cell types was carried out under identical growth conditions post-irradiation while the cell proliferation assays were carried out under the respective optimal growth conditions for the two cell types. That both assays showed the radio-resistance of the mammosphere cells further supports the idea that this reflects the intrinsic radiosensitivity of the cells rather than an artifact of the culture conditions.

We have now established several other facets of the response of mammospheres to endogenously and exogenously induced DNA damage. It is clear that mammospheres possess efficient base excision repair/SSBR, probably mediated by an elevated level of APE1. Although base excision and SSBR are largely responsible for cellular protection from endogenous oxidative damage, they also play a role in response to radiation and antineoplastic agents. For example, in breast cancer patients receiving the radiomimetic drug, anthracycline, lower APE1 expression was associated with a better pathologic response [56]. We also observed that mammospheres exhibit a similar DSBR response after irradiation as the monolayer cells in spite of the lack of H2AX phosphorylation, implying that mammospheres use a γH2AX-independent pathway for DSBR. The formation of 53BP1 and Rad51 foci in the irradiated mammospheres strongly suggests that both the NHEJ and HR pathways can be initiated, although further study will be required to fully elucidate the DSBR pathway utilization by these cells.

In terms of radiation-induced cell death, the most important pathway for MCF-7 cells appears to be replicative senescence but the mammospheres displayed a markedly reduced tendency to undergo senescence. The immediate molecular basis for the senescent behaviour can be attributed to the robust telomerase activity and negligible p21 expression. However, p21 expression is regulated by ATM through Chk2 and p53 [57], and thus implicit in our findings is that reduced Chk2 phosphorylation/activation may play a significant role in the senescence phenotype of MCF-7 mammospheres.

Finally, it is to be anticipated that cellular pathways utilized by cancer stem cells to enhance survival will depend on the genetic background of the stem cells. For example, MCF-7 cells are representative of cancer cells that do not readily undergo apoptosis, and thus enhanced survival of MCF-7 cancer-initiating cells is primarily dependent on down-regulation of the senescence pathway. A similar response may be expected in other cancer stem cells, such as those with inactivating mutations in the proline-rich domain of p53, which is required for p53-dependent transactivation of key apoptotic genes but not p21 [58,59]. On the other hand, avoidance of apoptosis is likely to play a more critical role in the survival response exhibited by cancer stem cells that possess potential functional apoptotic activity. This may explain the differences in DNA repair/cell cycle protein expression and post-translational modification seen between the current study and Bao and colleagues [20].

## Conclusions

MCF-7 CD24^-/low^/CD44^+ ^tumor-initiating cells, grown as mammospheres, have previously been shown to be more resistant to ionizing radiation than the general population of MCF-7 adherent cells. We have carried out a comprehensive comparison of the DNA damage/cell cycle and cell death responses of the two cell types. Enhanced survival of MCF-7 tumor-initiating cells in response to ionizing radiation is primarily dependent on an inherent down-regulation of the senescence pathway, which can be attributed to elevated telomerase activity as well as reduced p21 expression and pRb phosphorylation. Our data highlight the need to consider targeting proteins that regulate senescence in tumor-initiating cells, especially in tumor cells such as MCF-7 that do not readily undergo apoptosis.

## Abbreviations

APE1: apurinic/apyrimidinic endonuclease 1; ATM: ataxia telangiectasia mutated; bp: base pairs; DMEM: Dulbecco's eagle modified media; DNA-PKcs: DNA-dependent protein kinase catalytic subunit; DSBR: double-strand break repair; FCS: fetal calf serum; HR: homologous recombination; NHEJ: nonhomologous end-joining; PBS: phosphate-buffered saline; ROS: reactive oxygen species; RT-PCR: reverse-transcription polymerase chain reaction; SSBR: single-strand break repair.

## Competing interests

The authors declare that they have no competing interests.

## Authors' contributions

FKB and ARN carried out all the experiments. FKB, JRM and MW designed the experiments and prepared the manuscript.

## Supplementary Material

Additional file 1 **Isolation and identification of mammospheres**. **(a) **Formation of a 'dome' in a culture of MCF-7 monolayer breast cancer cells and **(b) **generation of non-adherent mammospheres after 14 days of cultivation visualized by light microscopy. **(c) **Example of CD24/CD44 expression in breast cancer cells grown in monolayer culture (left) and as mammospheres (right). Cells were incubated with phycoerythrin-labelled anti-CD24 and FITC-labelled anti-CD44 antibodies and analyzed by flow cytometry. **(d) **Overlay of the CD24 signals for the monolayer and mammosphere cells indicating the decrease in CD24 in the latter population (median values: 1036 for the monolayer cells vs 125 for the mammosphere cells). Click here for file

Additional file 2 **Cell-cycle analysis of unirradiated MCF-7 monolayer and mammosphere cell populations**. Cell cycle was determined by FACS analysis following propidium iodide staining. Open bars represent confluent monolayer cells, hatched bars represent monolayer cells in log-phase and solid bars represent mammospheres. Click here for file

Additional file 3 **Radiation-induced (a) 53BP1 foci and (b) Rad51 foci in MCF-7 monolayer and mammospheres cells**. Unirradiated cells or cells exposed to 5 Gy γ-radiation and then incubated at 37°C for different times, were fixed, permeabilized and immunostained with antibodies to either 53BP1 or Rad51. Click here for file
