# Supplementary figures and images for: Senescence evasion by MCF-7 human breast tumor-initiating cells
Source: Breast Cancer Res. 2010 Jun 2;12(3):R31. doi: 10.1186/bcr2583 (PMC2917024; doi:10.1186/bcr2583)

## Slide 1
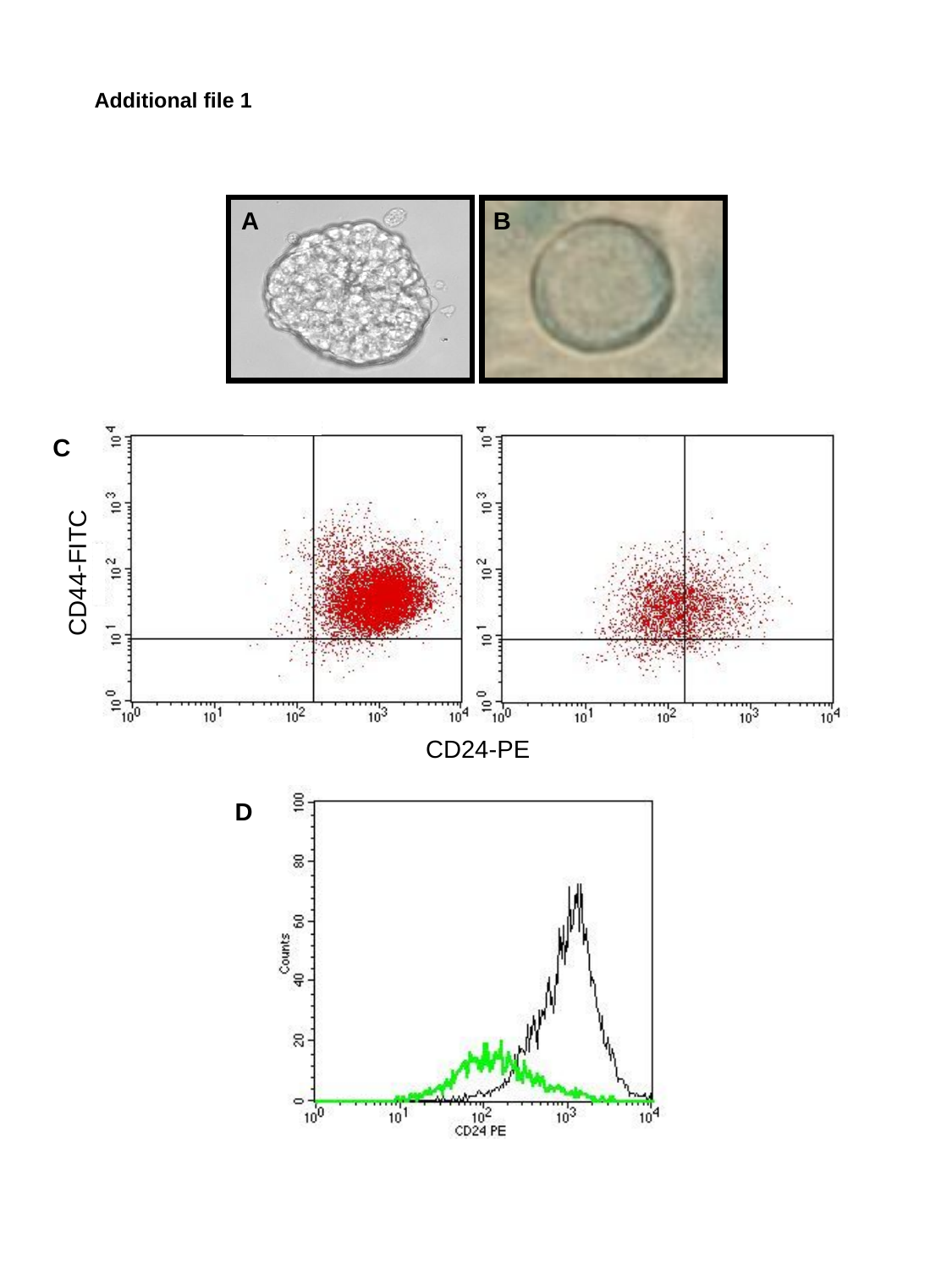

Additional file 1
A
B
C
CD44-FITC
CD24-PE
D

Supplement: Additional file 1 — Isolation and identification of mammospheres. (a) Formation of a 'dome' in a culture of MCF-7 monolayer breast cancer cells and (b) generation of non-adherent mammospheres after 14 days of cultivation visualized by light microscopy. (c) Example of CD24/CD44 expression in breast cancer cells grown in monolayer culture (left) and as mammospheres (right). Cells were incubated with phycoerythrin-labelled anti-CD24 and FITC-labelled anti-CD44 antibodies and analyzed by flow cytometry. (d) Overlay of the CD24 signals for the monolayer and mammosphere cells indicating the decrease in CD24 in the latter population (median values: 1036 for the monolayer cells vs 125 for the mammosphere cells). [file bcr2583-S1.PPT]

## Slide 1
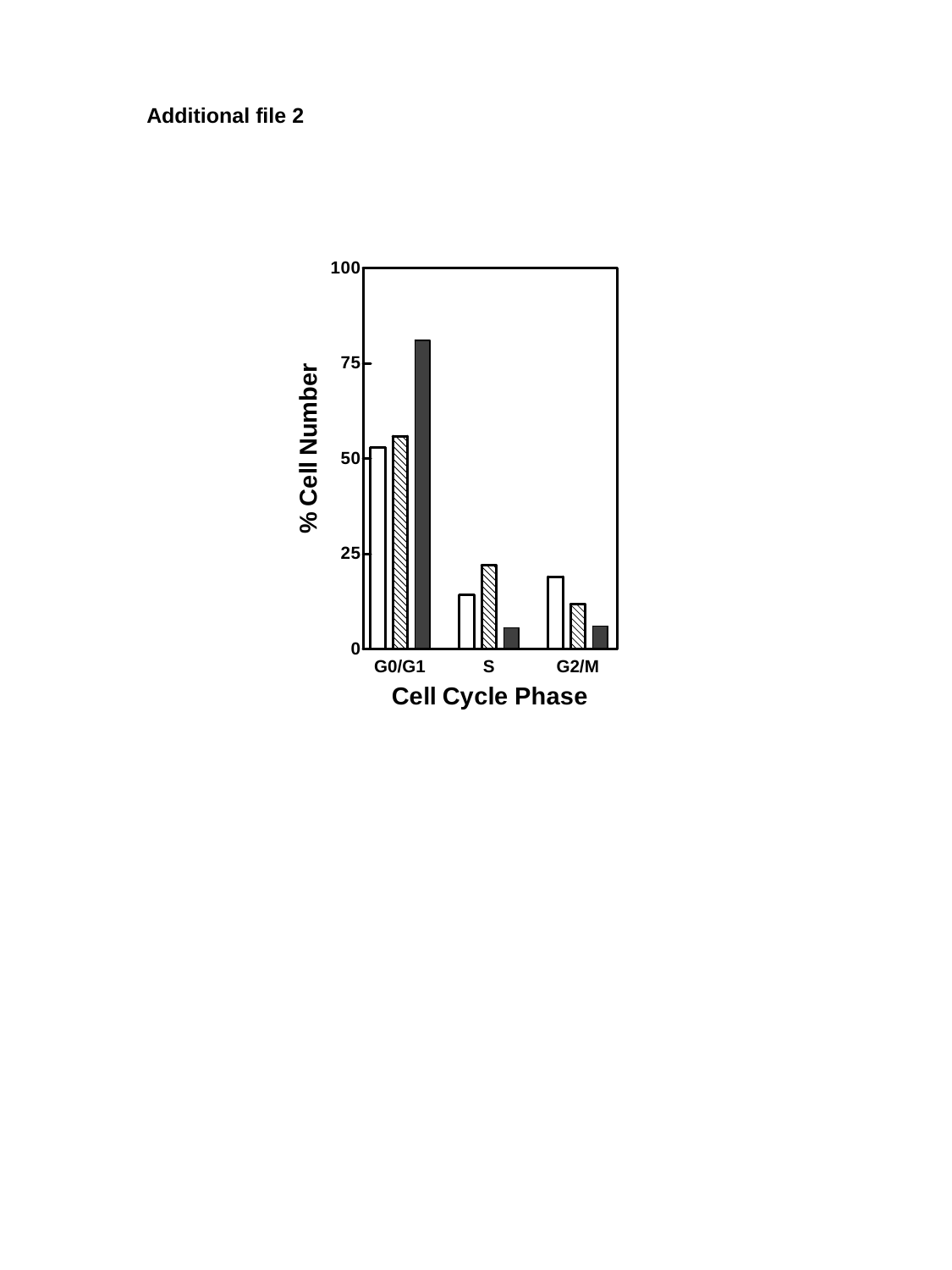

Additional file 2

Supplement: Additional file 2 — Cell-cycle analysis of unirradiated MCF-7 monolayer and mammosphere cell populations. Cell cycle was determined by FACS analysis following propidium iodide staining. Open bars represent confluent monolayer cells, hatched bars represent monolayer cells in log-phase and solid bars represent mammospheres. [file bcr2583-S2.PPT]
