# Supplementary material for: Senescence evasion by MCF-7 human breast tumor-initiating cells
Source: Breast Cancer Res. 2010 Jun 2;12(3):R31. doi: 10.1186/bcr2583 (PMC2917024; doi:10.1186/bcr2583)
Supplement: Additional file 3 — Radiation-induced (a) 53BP1 foci and (b) Rad51 foci in MCF-7 monolayer and mammospheres cells. Unirradiated cells or cells exposed to 5 Gy γ-radiation and then incubated at 37°C for different times, were fixed, permeabilized and immunostained with antibodies to either 53BP1 or Rad51. [file bcr2583-S3.PPT]

## Slide 1
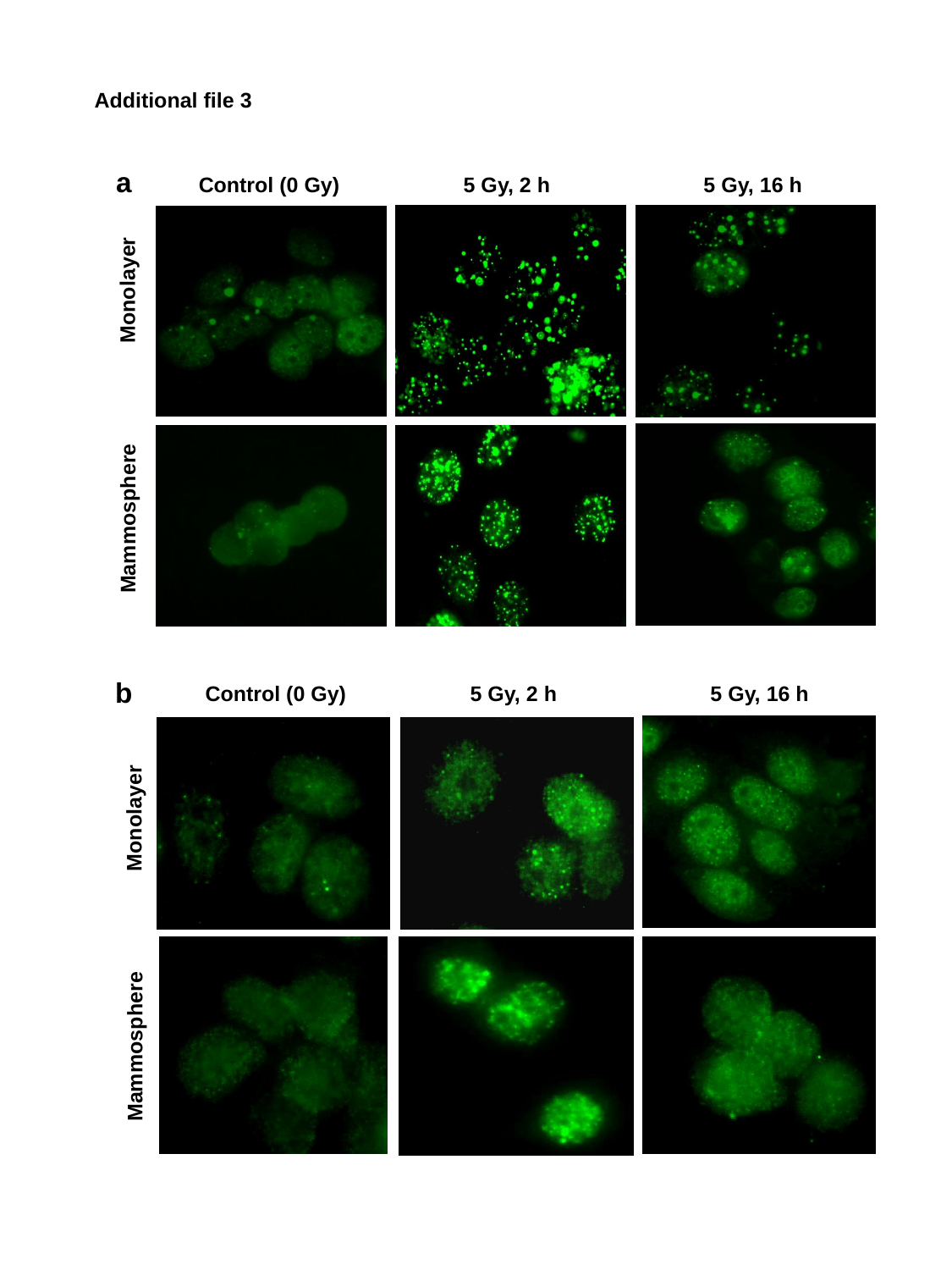

Additional file 3
a
Control (0 Gy) 5 Gy, 2 h 5 Gy, 16 h
Monolayer
Mammosphere
b
Control (0 Gy) 5 Gy, 2 h 5 Gy, 16 h
Monolayer
Mammosphere
